# Influence of Patient-Specific Head Modeling on EEG Source Imaging

**DOI:** 10.1155/2020/5076865

**Published:** 2020-04-03

**Authors:** Yohan Céspedes-Villar, Juan David Martinez-Vargas, G. Castellanos-Dominguez

**Affiliations:** ^1^Signal Processing and Recognition Group, Universidad Nacional de Colombia, Manizales, Colombia; ^2^Instituto Tecnológico Metropolitano, Medellín, Colombia

## Abstract

Electromagnetic source imaging (ESI) techniques have become one of the most common alternatives for understanding cognitive processes in the human brain and for guiding possible therapies for neurological diseases. However, ESI accuracy strongly depends on the forward model capabilities to accurately describe the subject's head anatomy from the available structural data. Attempting to improve the ESI performance, we enhance the brain structure model within the individual-defined forward problem formulation, combining the head geometry complexity of the modeled tissue compartments and the prior knowledge of the brain tissue morphology. We validate the proposed methodology using 25 subjects, from which a set of magnetic-resonance imaging scans is acquired, extracting the anatomical priors and an electroencephalography signal set needed for validating the ESI scenarios. Obtained results confirm that incorporating patient-specific head models enhances the performed accuracy and improves the localization of focal and deep sources.

## 1. Introduction

Electroencephalography (EEG) and Magnetoencephalography (MEG) recordings are widely used as noninvasive neuroimaging techniques to describe the dynamics of brain activity, driving to a better understanding of cognitive processes and neurological diseases in the human brain. Nonetheless, recorded EEG/MEG signals from each scalp electrode are affected by the volume-conducted activity coming from multiple sources spatially dispersed in the brain cortex [1]. Although signals are directly influenced by the conductivity patterns of each head tissue, many studies concentrated on the effects of realistic head modeling on the EEG modality since MEG is assumed to be less affected by uncertainties inherent to the experimentally determined conductivity values of the different conductive compartments [2]. To date, several strategies have been devised to improve the spatial representation of local interactions between brain sources generating the recorded scalp potentials. Among others, the following techniques are worth mentioning in electromagnetic studies: removing or minimizing the unwanted nonbrain signals or artifacts [3, 4], rereferencing of acquired EEG data [5], and enhancing the source space analyses or source estimation models (termed electromagnetic source imaging (ESI)) that are employed to estimate the synchronously active neural sources, which generate the electrical potentials measured over the scalp [6]. The conductivity of brain tissues is not only essential for ESI tasks but also a fundamental reflector of the brain functional changes like in deep brain stimulation [7] and transcranial electrical stimulation that is a useful tool for the neurophysiological characterization and diagnosis of several neurological disorders, eliciting changes in cortical excitability [8]. But to increase the precision of estimated brain sources and to provide target-specific stimulation, a personalized pipeline is required for an accurate head model generation as realistic as possible [9], which is much more complex and must consider tissue conductivities and the individual shape of the compartments with different electrical conductivity.

A baseline approach to constructing realistic head models is to build approximate representations from measures of head geometry and the electrical conductivity field. This information can be extracted from MR scans collected for quantitative brain analysis [10] or using phase-based conductivity mapping that relies on the fact that the conductivity of material primarily affects the phase of the magnetic field [11]. So a realistic conductivity model is built by solving the potential flow equations (termed the forward problem) employing the segmented head volume. Using a regular voxelization of head volumes, the finite difference method (FDM) offers a solution for the forward problem holding a grid partition that directly adapts to the existence of discrete structural MRI data. Thus, once the procedures of MRI register and segmentation are carried out, an accurate head conductivity volume can be obtained, encoding the individual structural characteristics extracted from the available patient-specific data. The main reason for conducting segmentation is that tissue differentiation helps to assign the appropriate conductivity values, as explained by [12].

Regarding the source space of ESI solutions, a single mesh of the gray matter typically contains thousands of current dipoles (sources), which are to be placed in fixed locations over the brain [13]. Nonetheless, sources of electrical activity must be confined to the surface of the cerebral cortex (i.e., the gray matter) since it is the most representative location of cortical activity using a tessellated mesh of the cortical surface, resulting in a challenging task that commonly needs manual intervention [14]. Thus, the most common approach for creating the source space using volumetric head conductivity solutions like FDM is to warp a standard source template into the subject's brain, creating a subject-specific model. Still, warping a cortical mesh from a template tends to produce nonexact correspondences of sulci across the brain model, yielding important errors of source reconstruction in cases of low-quality MRI scans. Another aspect to consider is that head tissue conductivity specificities differently influence the sensitivity of source reconstruction methods, depending on whether they are based on focal (dipole) sources or those for reconstructing widespread brain activity (based on goal function scans). This influence is especially strong for skull and skin compartments, yielding source localization errors in the range of centimeters, as summarized in [15].

In some situations, however, the structural MRI data collected from individuals may be unavailable for implementing ESI solutions. Another possible approach to constructing models is to perform a pattern over a precalculated conductivity head model than can be already segmented. Due to the considerable time required to calculate individual head models, however, the use of a generalized atlas model is the most desirable solution that encodes the normal anatomical variability in the population under study as accurately as possible. Still, ESI solutions may benefit from additional information on individual patient-specific conductivity head models. In this line of research, we analyze the influence of conductivity head models regarding the dependence between structural MRI data and demography of populations. To accomplish this goal, we employ a generalized atlas that can be built by averaging several individual head models across a large population. Thus, we employ the New York head model segmentation that is generated from 152 subjects, holding a five-layer volumetric partitioning [16]. Although this approach is inexpensive because it avoids the acquisition of MRI scans, the resulted standard model is far from being individually representative of the wide variety of subjects/populations. A step further is to construct a head model atlas by averaging the geometry information of the objective population under examination. However, despite the level of anatomical detail achieved by the atlas head models, the wrong geometry approximations because of the averaging procedure may result in significative source localization errors [17]. Lastly, head models are calculated individually for each patient, encoding the particular information on each subject in the conductivity head model.

Nevertheless, the use of individually defined forward models can be mandatory in several medical applications that demand a proper assisted diagnosis and adequate interpretation of patient states from the involved neurophysiological data [18–20]. In this regard, the combination of both approaches to enhance the model of brain structure differently implies inter- and intraobserver variability, not always decreasing enough the impact of uncertainty in the inherent geometrical complexities. Hence, within the forward problem formulation, there is a need for constructing individually defined source spaces that enable the EEG source localization to be more accurate and consistently enhance the interpretability of source reconstruction studies.

Here, we present a methodology for improving the EEG source localization performance that incorporates tissue information and prior knowledge, relying on realistic patient-specific data and endeavoring to enhance the model of brain structure in the forward problem formulation. The methodology relies on two well-known ESI methods, namely, Low-Resolution Tomography (LORETA) and Multiple Sparse Priors (MSP), both of them implemented under a Bayesian formulation. For validation purposes, we calculate a realistic patient-specific head model for the following three structural complexities: Firstly, we perform the baseline three-layer segmentation holding the scalp, skull, and brain tissues. Then, we also include CSF, as suggested in [21]. Finally, we accomplish a five-layer segmentation, including the scalp, skull, CSF, gray matter (GM), and white matter (WM). These three levels of complexity allow analyzing the influence of conductivity modeling complexity on the performance of EEG source imaging problems. Obtained results prove that incorporating patient-specific head models enhances the performed ESI accuracy, improving the localization of focal and deep sources. The agenda is as follows: In Section 2, we outline the proposed methodology for creating individually defined forward models, and we describe the employed ESI methods under a Bayesian formulation. Further, Section 3 describes the experimental setup designed for comparing several scenarios of individualized forward models with real EEG data. In Section 4, we discuss the interpretation of the obtained results, remarking our contribution in Section 5.

## 2. Methods

### 2.1. EEG Forward Problem and Numerical FDRM Solution

The EEG forward problem entails calculating the electrical potentials of the scalp surface for a single current source, given the electrical conductivity and geometry of the head volume. Therefore, as the relevant frequencies of the EEG spectrum are below 100 Hz, the quasistatic approximation of Maxwell's equations is used to estimate the potentials over the scalp. These potentials are generated by an electric current in the brain, assuming an inhomogeneous medium with isotropic conductivity. Thus, the forward problem requires solving the Poisson equation as follows:

(1) $$\begin{array}{c} \nabla \cdot \left(\Sigma \left(x,y,z\right)\nabla \Phi \left(x,y,z\right)\right)=-\nabla \cdot J\left(x_{\iota_{f}},y_{\iota_{f}},z_{\iota_{f}}\right), \end{array}$$

where *J*(*x*_*ι*_*f*__, *y*_*ι*_*f*__, *z*_*ι*_*f*__) ∈ ℝ is the electric current density in the specific location {*x*_*ι*_*f*__, *y*_*ι*_*f*__, *z*_*ι*_*f*__} ∈ *Ω*. Moreover, *Φ*(*x*, *y*, *z*) ∈ ℝ is the unknown potentials, *Σ*(*x*, *y*, *z*) ∈ ℝ^+^ is the spatially varying conductivity function, and {*x*, *y*, *z*} is any location of the head region *Ω*.

Solution of Equation (1) requires defining the boundary conditions properly between two compartments (head layers) with different conductivity values, which are separated by the interface Γ_*i*,*j*_. Thus, the boundary conditions state that all charges, leaving one compartment with conductivity *Σ*_*i*_, must be transferred to the other compartment with conductivity *Σ*_*j*_ (termed the Neumann statement):

(2) $$\begin{array}{c} \;\left(\Sigma_{i}\nabla \Phi_{i}\right)\cdot {\hat{e}}_{n}=\left(\Sigma_{j}\nabla \Phi_{j}\right)\cdot {\hat{e}}_{n}, \end{array}$$

where ${\hat{e}}_{n}$ is a vector normal to the interface surface Γ_*i*,*j*_, and *Σ*∇*Φ* represents the charge. As a special case, because of the very low conductivity of air, no current flows out of the human head (homogeneous Neumann statement). Therefore, the current density at the head surface boundary Γ_*Ω*_ is expressed as ${\left.\left(\Sigma \nabla \Phi \right)\cdot {\hat{e}}_{n}\right|}_{\Gamma_{\Omega }}=0$.

Furthermore, solving Equation (1) also requires a numerical framework, allowing a piecewise solution of the partial differential equations in a volumetric domain that properly deals with realistic head models with irregular boundaries [22]. For fulfilling the requirements mentioned above, we employ the approach developed in [23] that relies on the method introduced earlier in [24], in which the finite difference formulation of the Laplace equation (see [25]) is extended to the Poisson equation equipped with dipole current source generators. The proposed solution remains valid everywhere in a piecewise inhomogeneous isotropic medium under the assumption that boundaries Γ_*i*,*j*_ between neighboring compartments are smooth enough. Following the FDRM approach, Equation (1) is discretized by a 19-point stencil with 8 voxels that share the same vertex *ϕ*_0_^*j*^, enabling a linear formulation for the vertex *ϕ*_*i*_^*j*^ in the stencil *S*_*j*_ around *ϕ*_0_^*j*^, as follows:

(3) $$\begin{array}{c} \overset{18}{\underset{i\in S_{j}}{\sum }}\alpha_{i}^{j}\phi_{i}^{j}-\left(\overset{18}{\underset{i\in S_{j}}{\sum }}\alpha_{i}^{j}\right)\phi_{0}^{j}=\iota_{f}, \end{array}$$

where *ϕ*_*i*_^*j*^ ∈ ℝ is the scalar-valued potential at the *i*-th neighbor vertex of the *j*-th node in the stencil *S*_*j*_. *ι*_*f*_ ∈ ℝ is the dipole current, and *α*_*i*_^*j*^ ∈ ℝ is the FDM coefficients depending on the conductivity value *Σ* and the internode distance [25].

We propose to solve the system in Equation (3) only within the *Ω* domain, holding the interface Γ_*Ω*_, that fully contains the significant potential unknowns represented by *ϕ*_*i*_. Also, Equation (3) yields a linear system that is solved using the BiCG stabilized solver with iLU preconditioning [26]. However, every single forward calculation requires a high computational burden. To overcome this issue, precalculated reciprocity potentials are employed to speed up the computation of associated inverse solutions. Thus, we calculate a lead field matrix **L** ∈ ℝ^*C*×*D*^ for a given electrode disposition at *C* channels and source space with *D* dipoles located on the cortical surface with a fixed orientation perpendicular to it.

### 2.2. EEG Source Imaging

Given the lead field matrix **L** that is obtained from the forward model, the distributed ESI methods represent the electromagnetic field measured by EEG by the following linear model:

(4) $$\begin{array}{c} Y=LJ+\Xi , \end{array}$$

where **Y** ∈ ℝ^*C*×*T*^ is the EEG data measured on a set of *C* sensors at *T* time points and **J** ∈ ℝ^*D*×*T*^ is the amplitude of *D* current dipoles distributed through the cortical surface with a fixed orientation perpendicular to it. Moreover, we assume that the EEG data is corrupted by noise **Ξ** ∈ ℝ^*C*×*T*^ with covariance cov(**Ξ**) = **Q**_*Ξ*_, with **Q**_*Ξ*_ ∈ ℝ^*C*×*C*^. Assuming that sources are a zero mean Gaussian process with prior covariance cov(**J**) = **Q**, with **Q** ∈ ℝ^*D*×*D*^, brain activity estimation is carried out within a Bayesian framework by solving the maximum-a-posteriori problem in the form [27]

(5) $$\begin{array}{c} \hat{J}=\underset{J}{\text{argmax}}\left\{p\left(J\;|\;Y\right)\right\}=\underset{J}{\text{argmax}}\left\{p\left(Y\;|\;J\right)p\left(J\right)\right\}. \end{array}$$

The optimization problem in Equation (5) yields the estimate $\hat{J}=QL^{Τ}{\left(Q_{\Xi }+LQL^{Τ}\right)}^{-1}Y$ that depends on the sensor and source covariance matrices. The sensor noise covariance is set as **Q**_*Ξ*_ = exp(*λ*_*Ξ*_)**I**_*C*_, where **I**_*C*_ ∈ ℝ^*C*×*C*^ is an identity matrix and exp(*λ*_*Ξ*_) ∈ ℝ^+^ is a hyperparameter modulating the sensor noise variance [28]. Also, we use two different approaches to build the source covariance matrix [29]:

- *Low Resolution Brain Electromagnetic Tomography (LORETA)*. The source covariance matrix, holding a Laplacian operator Δ ∈ ℝ^*D*×*D*^, that is aimed at representing the groups of neurons with synchronized activation, seeking smoothness [30]:

(6) $$\begin{array}{c} Q=\exp\left(\lambda_{1}\right){\left(L^{Τ}\Delta^{Τ}L\Delta \right)}^{-1} \end{array}$$

(ii) *Multiple Sparse Priors (MSP)*. The source covariance matrix is built a sum of *P* patches, each one reflecting a single potentially activated region of cortex that is weighted by its respective hyperparameter as follows [31]:

(7) $$\begin{array}{c} Q=\underset{p\in P}{\sum }\exp\left(\lambda_{p}\right)Q_{p} \end{array}$$

Further, we use the so-termed free energy to estimate the hyperparameter set as follows [32]:

(8) $$\begin{array}{c} F=\frac{T}{2}tr\left(\Delta^{-1}S\right)-\frac{T}{2}\ln\left|\Delta \right|-\frac{CT}{2}\ln2\Pi -\frac{1}{2}{\left(\mu -\eta \right)}^{T}\Omega^{-1}\left(\mu -\eta \right)+\frac{1}{2}\ln\left|Y\Omega^{-1}\right|, \end{array}$$

where Δ = **LQL**^*Τ*^ + **Q**_*Ξ*_ is the estimated model covariance, with Δ ∈ ℝ^*C*×*C*^, and **S** ∈ ℝ^*C*×*C*^ is the measured data covariance. Besides, *μ* and *η* ∈ ℝ*P* + 1 × 1 are vectors of prior and posterior hyperparameter means, and **Ω** and **Y** ∈ ℝ^*P*+1×*P*+1^ are the prior and posterior hyperparameter covariance matrices, respectively. Since the free energy estimated in Equation (8) is a trade-off between the model accuracy (the first two terms) and model complexity (the last two terms), it is commonly used to measure the source reconstruction performance [33].

## 3. Experimental Framework

**Figure 1:**
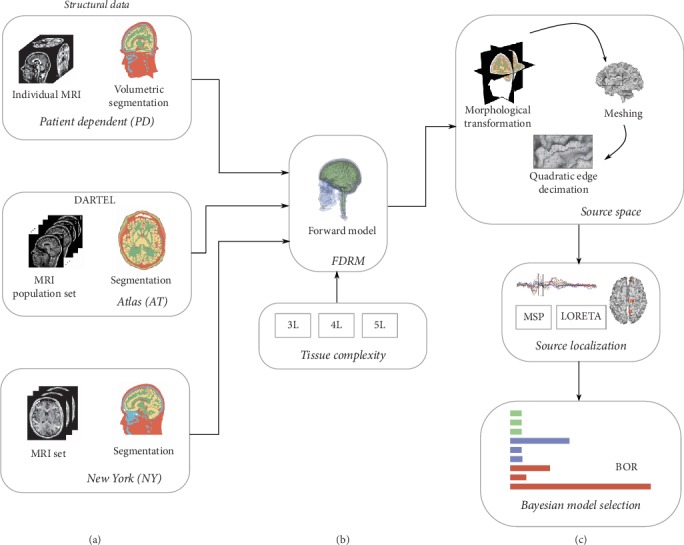
Schematic methodology representation for enhancing the brain tissue model tested within the EEG forward problem formulation. The figure shows the proposed individually defined head modeling, including patient-dependent structural MRI, individual MRI segmentation, FDRM, and individual source space modeling. The middle and bottom boxes of (a) show the comparative structural information, namely, atlas (AT) and New York (NY). The top middle panel of (b) shows different tissue complexities (3L, 4L, and 5L). The remaining panels of (c) show the source localization and the performance measure.

We develop an individually defined head modeling process that includes the computing of the subject-specific cortical mesh and also the enhancement of the model of the brain structure, as shown in Figure 1. In the last case, we evaluate two approaches to enhance the model of the head structure in the EEG forward problem formulation: (i) Promoting patient-dependent data by the gradual incorporation of practical knowledge of the brain tissue morphology, concerning each patient. For comparison purposes, the anatomical structure priors are extracted individually for each one of the following cases of image data: a standardized MRI template, a demographic population atlas, or a set of patient-specific MRI scans. (ii) Constructing a more precise volumetric tissue model by augmenting the number of segmented brain tissues. Namely, we contrast the following configurations of tissue model complexity: three-layer arrangement (noted as 3L), including the homogenized brain compartment, skull, and scalp; 4L, adding CSF to the 3L configuration; and 5L that divides the 4L brain model into white matter and gray matter. Thus, for each image data and configuration of tissue model complexity, a head model is computed. As regards the differences in head model segmentation, the atlas is partitioned using probabilistic maps available in the averaging group process. By contrast, the individual segmentation is performed using the FieldTrip routine. Although both segmentation procedures in Figure 1 are not identical, each one preserves mainly the brain structure of individuals in the conductivity volume.

Evaluation of the proposed methodology to enhance the head tissue model is accomplished within the Bayesian formulation of the EEG inverse problem, where the MSP and LORETA methods are carried out as the ESI solution.

### 3.1. Neuroimage Datasets

The brain image data were acquired from 25 children within an age range between 5 and 16 years old, having two sociocultural levels (high-medium and low-medium). All patients were randomly selected from the preschool, elementary, and secondary courses at a few private and public schools of Manizales city. For legal purposes, the ethical committee of *Universidad Autónoma de Manizales* approved the study, and the children's parents agreed to participate in the research through written permission. According to the children's historical data, the exclusion criteria were established for mental retardation, individuals with neurological antecedents (history of head trauma, epilepsy, and related), or referring psychiatric disorders (psychiatric hospitalization history, autism, and similar).

From each child, two brain image datasets were acquired: an MRI collection supplying the anatomical priors of the brain tissues and an *EEG/ERP* set that is employed for validation of the EEG source imaging performance. Further, we rely on the fact that the EEG electrode placement of the volumetric forward model has an important role in ESI solutions. Therefore, we perform a fiducial-based similarity transformation to align the EEG electrodes to the head volume. Afterward, we project each electrode position towards the center direction of the head volume to ensure that every single electrode is surrounded by scalp voxels but guaranteeing that the electrode voxel is not surrounded by air.

- *MRI*. A set of T1-MRI scans are acquired from the same 25 children under study, employing a 1.5T General Electric Optima MR360 scanner with the following parameters: 1 mm × 1 mm pixel size, *T*_*R*_ = 6, *T*_*E*_ = 1.8, *T*_*I*_ = 450, and sagittal slices of 256 × 256 size and 1 mm spacing. For each child, three scans are performed to be further averaged (using the free surfer suite), yielding a single representative brain MRI to provide an enhanced signal-to-noise ratio
- *EEG/ERP*. The brain activity data were obtained following an oddball experimental paradigm for cognitive evoked potentials with rare visual stimuli, where each evoked stimulus lasted 130 ms, while the time delay between the onsets of two consecutive stimuli was 1 s. During each stimulation, the subjects had to pay attention to the rare stimulus (termed *target*) and count their occurrence, ignoring the presence of remaining stimuli (*nontargets*). The nontarget stimuli were displayed on 80% of the trials, whereas the target stimuli on 20% of the remaining trials, resulting in approximately 160 nontarget stimuli and 40 target stimuli. The EEG recordings were collected using 19 electrodes symmetrically placed in the standard positions of the international 10-20 system, operating a single *Easy III EEG amplifier* (Cadwell). Data were subsampled at 250 Hz and segmented in 1 s epochs, which were averaged separately over each subject and stimulation condition. As a result, two ERPs were obtained following the different stimulus conditions for each subject, namely, visual target (V-T) and visual nontarget (V-nT).

### 3.2. Head Models Integrating Brain Structure Priors

For incorporating anatomical priors of the brain tissues in the EEG forward problem, we evaluate the ESI performance individually over the following head models:

- *Template-Based Head Model (NY)*. As a standardized version of volume conductor models, we use the New York (NY) head that is frequently used in neuroimage studies whenever an individual MRI is not available. NY allows segmentation of one symmetric head template (ICBM-152 v2009) into two tissue types (gray matter (GM) and white matter (WM)) and partition of another symmetric template (ICBM-152 v6) into nonbrain tissues (CSF, skull, and scalp). Also, the segmentation of the lower head part is extracted from an additional head template averaged over 26 subjects which provided the data collection. Thus, the segmented head tissues, together with the extracted lower part, are compounded into the NY head model
- *Atlas (AT) Head Model*. An anatomical brain atlas is built to provide a more precise demographic tissue information about a concrete population sample. So we build a generic atlas from the obtained 26 representative MRI scans, employing the Diffeomorphic Anatomical Registration Through Exponentiated Lie (DARTEL) algorithm that generates a set of customized templates for the considered brain tissues [34]. The use of DARTEL nonlinearly transforms all individual probabilistic partitions, initially provided by the Statistical Parametric Mapping (SPM) MATLAB Toolbox, merging them into a single template. The registration procedure is conducted during a fixed number of iterations for increasing the template crispness. To construct the atlas from all patients, including all tested tissues, DARTEL is applied using the following default parameters: linear elastic energy regularization, Levenberg–Marquardt optimization, and six outer iterations
- *Patient-Dependent (PD) Head Model*. This model of prior information includes patient-dependent structure data individually and tends to provide more specific knowledge of the geometry of each child's brain, performing an individual MRI segmentation of all considered brain tissues separately. Nevertheless, the resulting segmentation, which is accomplished using the FieldTrip pipeline [35], may contain some anatomic errors, like WM patches surrounded by a skull or skull exposed outside the scalp. Therefore, the segmentation is corrected to ensure that the GM tissue covers WM entirely, being contained by CSF at the same time. It is worth noting that the brain segmentation procedure includes the neck area extracted for each patient [36]. As regards the differences in head model segmentation, the atlas is partitioned using probabilistic maps available in the averaging group process. By contrast, the individual segmentation is performed using the FieldTrip routine. Although both segmentation procedures are not identical, each one preserves mainly the brain structure of individuals in the conductivity volume.

### 3.3. EEG Source Imaging in Forward Solution

For accurate localization of very focal sources, we aim to construct individually defined cortical meshes. Thus, to obtain realistic modeling of the sulci and gyrus of the brain, we propose to apply morphological operators over the WM volumetric segmentation, reproducing better the activity of a cortex layer that belongs to individual voxels within the volumetric regular FDM voxelization. This assumption is supported by the fact that brain activity is mostly characterized by the distributed current sources and sinks located within the tissue of evoking source areas (e.g., grey matter) [37].

**Figure 2:**
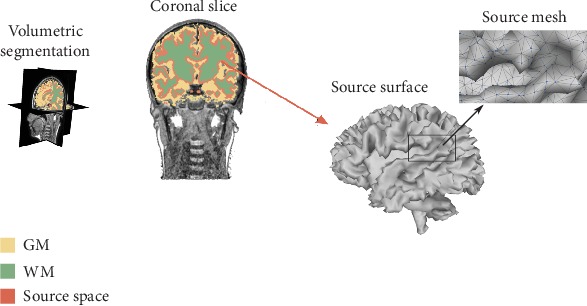
Source space generation trough individually defined cortical meshes using morphological operators.

The schematic representation of the proposed methodology for producing the source space is presented in Figure 2. Thus, we compute a neocortex surface mesh having close to 10.000 vertexes to provide enough resolution (in fact, the mesh from initial data has more than 2 × 10^5^ vertexes, which are downsampled using a quadratic edge decimation). Further, every single vertex becomes a source position so that the triangulation procedure results in a connectivity map, containing the information of the spatial relationship between the neighbor sources and every single voxel.

Based on the generated source space, then, the lead fields are calculated from the performed segmentation of brain tissues for the tested head model configuration (3L, 4L, and 5L). Besides the geometrical complexity, the volume conduction model must specify the conductivity distribution of the modeled tissue compartments. Here, we use the consistent conductivity values reported for the examined brain tissues as follows [12]: scalp = 0.43 S/m, skull = 0.008 S/m, CSF = 1.79 S/m, GM = 0.33 S/m, and WM = 0.14 S/m. Lastly, the EEG electrodes are coregistered to the segmented scalp surface, using the FieldTrip toolbox and allowing to estimate the volumetric forward models over a uniform volumetric space of 1 × 1 × 1 mm. Due to the high computational burden demanded by FDM techniques, we perform a reciprocity precalculation of the lead field matrix of the EEG channels to speed up the needed forward computations.

**Figure 3:**
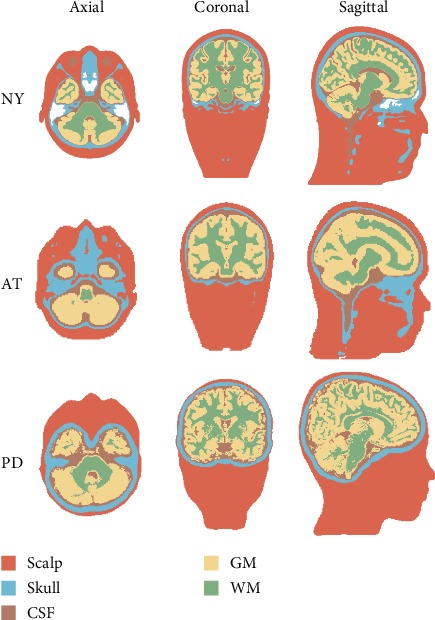
Exemplary of five-layered segmentation performed for the contrasted head models to incorporate prior information into the EEG forward model formulation.

Figure 3 displays some examples of the implemented segmentation of the examined head models incorporating prior information. Note that all evaluated head models in the individually defined forward modeling are computed using the EEG forward solution that we developed in [23].

### 3.4. Validating Scenarios of EEG Source Imaging

In accordance with the evaluated approaches to enhance the brain tissue model, we explore the following nine scenarios for evaluating the direct EEG problem: 3L-NY, 4L-NY, 5L-NY, 3L-AT, 4L-AT, 5L-AT, 3L-PD, 4L-PD, and 5L-PD.

In all scenarios, the source reconstruction of visual stimuli is validated using two ESI solutions: LORETA and MSP. In the former case, a Laplacian operator Δ is included to represent groups of neurons with synchronized activation (see Equation (6)), modeling spatial coherence. In the case of MSP, the priors, needed to compute the set of covariance components, are constructed as a sum of patches, each one reflecting one potentially activated region of cortex weighted by the respective hyperparameter (see Equation (7)). For either ESI solution, priors are implemented by the Statistical Parametric Mapping software package(SPM12), fixing the number of covariance components to 1024 for MSP, which are formed by sampling from evenly spaced columns of the coherence matrix as to cover the cortical surface entirely. Besides, the MSP hyperparameters are tuned by maximizing the free energy through the Restricted Maximum Likelihood approach under a greedy search algorithm.

During validation, we employ the experimental framework and metrics proposed in [38], developed for solving problems of group-level Bayesian model selection (BMS) in tasks that involve neuroimage and behavioral data. This approach is based on the free energy as an approximation to the Bayesian log evidence, yielding a reliable measure that shows which model is more probable to generate the available data [21]. To this end, we compare the free-energy values of the inverse solutions performed by subjects, elicited by each stimulation condition. Then, to measure the statistical risk of performing group BMS problems, the log group Bayes factor (*ρ*_*L*_ ∈ ℝ), the expected likelihood (*ρ*_*K*_ ∈ ℝ^+^), and the Bayesian omnibus risk (BOR, *ρ*_*B*_ ∈ ℝ^+^) are computed. In the log group Bayes factor, one model can be chosen in favor of another whenever there is a difference higher than the a priori fixed value. Here, if BOR is smaller than 0.25, the best model selected by the exceedance probability becomes trustworthy. Of note, the applied metrics estimate the probability of EEG data generating the sources, instead of calculating the accuracy as a distance from a âĂIJtrueâĂİ source to its inverse position.

## 4. Results

### 4.1. Performed Source Imaging Using Anatomic Structure Priors

**Figure 4:**
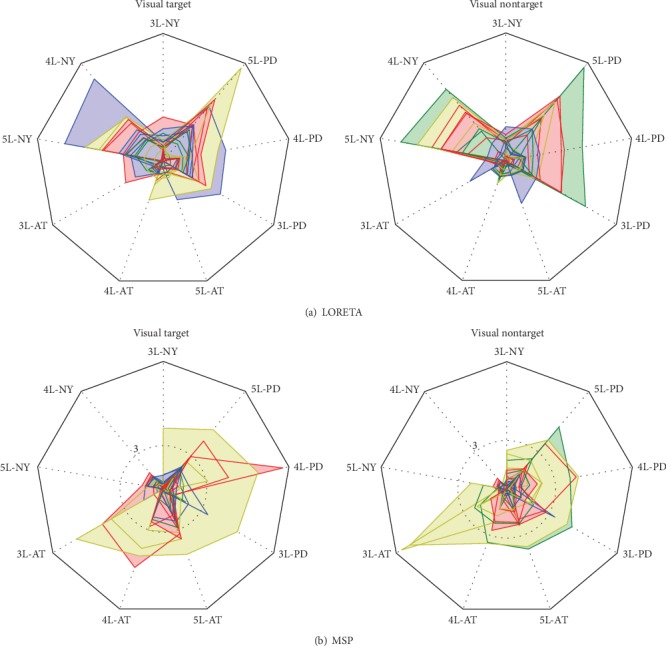
Radar chart showing the *ψ* values achieved by both ESI solutions for each scenario and patient. The gray circle is not drawn in the LORETA chart since the difference of patients for each image data and configuration of tissue model complexity is much lower than 3 points.

With the purpose of a better data visualization, all values of log Bayes factor *ψ* are represented through radar charts that display the source imaging performance data in a radial pattern, as shown in Figure 4. The star chart stacks the string data performed by each head model, subtracting the smallest free-energy value (i.e., the worst model) achieved through the corresponding stimulus condition. Therefore, the higher this computed factor, the better the model performs the EEG source reconstruction [39]. Note that each colored string depicts the performance obtained by a single subject, where the gray circle is drawn for the cases of significative Bayes factor, having differences that are greater than three points.

Figure 4(a) displays the achieved performance by LORETA source imaging solution, showing that the testing scenarios of NY produce the lowest values of *ψ* regardless of the considered stimuli. At the same time, the AT head models perform the worst. In the case of the MSP solution, Figure 4(b) shows that all testing scenarios of NY models produce the lowest values of *ψ* regardless of the considered stimuli. Concerning the AT models, the radar charts also show that the reconstruction accuracy improves, meaning that the inclusion of structural priors using the study population enhances the tissue model. However, the highest values of *ψ* are achieved by the PD head models, resulting in the best model for incorporating information about head tissues in the EEG forward problem. Another aspect to consider is the variability provided by each ESI solution for every patient. Thus, although MSP produces fewer outliers than the LORETA solution does, it is hard to select the best scenario during validation of the individually defined forward modeling.

### 4.2. Bayesian Model Selection of EEG Source Imaging Method

The BMS for group studies is employed, aimed at generalizing the performance results achieved by the examined ESI solutions across the whole patient set. Thus, BMS identifies the best source reconstruction through a random effect analysis, assuming that each subject is drawn from a large population of subjects so that his response represents an independent sample from the overall distribution [40]. We conduct BMS through the expected posterior probability and Bayesian omnibus risk (BOR), comparing the log model evidence for each reconstruction at the group level.

**Figure 5:**
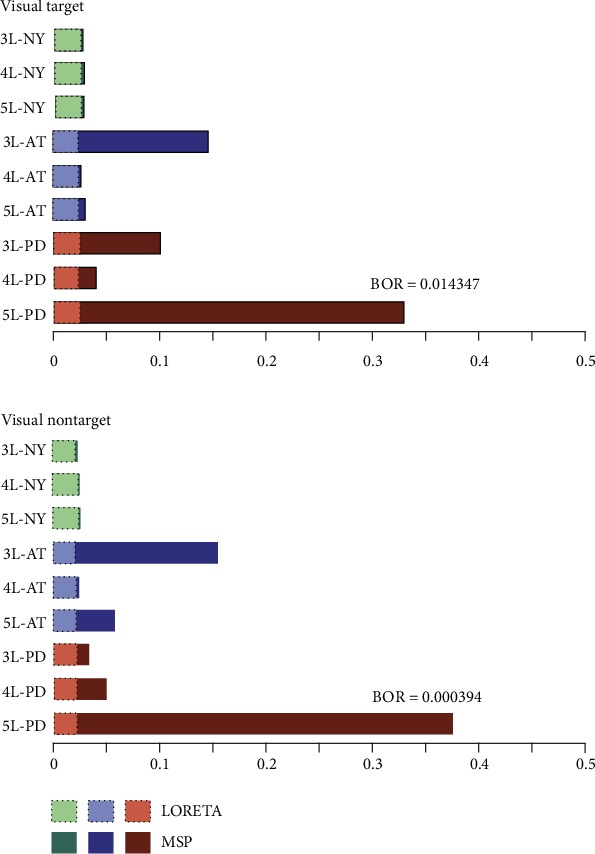
Results of Bayesian model selection shown as the expected posterior probability and Bayesian omnibus risk assessed by each testing scenario of a brain tissue model. LORETA and MSP are the contrasted ESI solutions.

In turn, Figure 5 presents the performed results, comparing together all the testing scenarios of MSP and LORETA solutions. It is worth noting that the confidence in the expected posterior probability is adequate for the considered visual stimulus (BOR lesser than 0.25).

On the other hand, MSP has better results than LORETA in all stimulus conditions despite the tested scenario of the head model, making obvious the superiority of the former solution regarding the achieved reconstruction accuracy. This effect may be explained by relying on the fact that the LORETA solution tends to fail in detecting deep sources. Furthermore, the deeper the actual source, the more blurred is the current density estimated by LORETA. On the contrary, the MSP algorithm embraces the entire cortical surface (both focal and deep), identifying the patches that better reproduce the neural source distribution, thus enabling a more accurate reconstruction of the superficial and deep sources.

**Figure 6:**
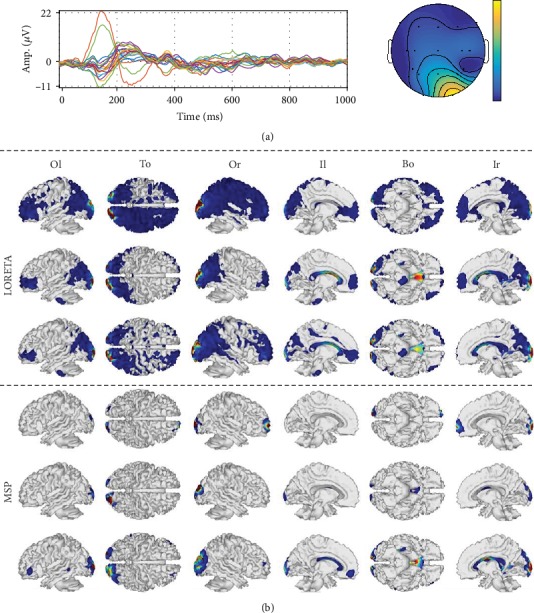
Achieved source reconstructions with NY models for both ESI methods. (a) Sensor space: ERP and topographic map. (b) Reconstructed activity. Views: Or: outside right; Ol: outside left; To: top; Bo: bottom; Ir: inside right; Il: inside left.

Further, Figure 6 displays the considered ESI solutions performed for a randomly selected patient, allowing to explain the above-described effect of different accuracies on the deep reconstructed sources. Besides, the NY head models are implemented due to the simplicity of their incorporated priors, having the lowest influence on source reconstruction. The obtained source reconstruction reveals that LORETA spreads the activity over the entire cortical mesh, although the identified areas that prevail tend to be less scattered as the number of modeled tissues increases. On the contrary, MSP focuses the most on the visual-processing-related areas (like the visual cortex in the posterior brain area), becoming more evident as the number of tissues increases.

Consequently, due to the shown outstanding superiority, we further consider the MSP solution in the following procedures. With the purpose of a better data visualization, all values of the log Bayes factor, *ψ*, are represented through the radar charts that display the source imaging performance data in a radial pattern as shown in Figure 4. The star chart stacks the string data performed by each head model, subtracting the smallest free-energy value (i.e., the worst model) achieved through the corresponding stimulus condition. Therefore, the higher this computed factor, the better the model performs the EEG source reconstruction [39]. Note that each colored string depicts the performance obtained by a single subject, where the gray circle is drawn for the cases of significative Bayes factor, having differences that are greater than three points.

### 4.3. Comparison of Brain Tissue Models

For the MSP source imaging, we compare the enhancing scenarios of the brain tissue model through the explained log model evidence at the group level.

**Figure 7:**
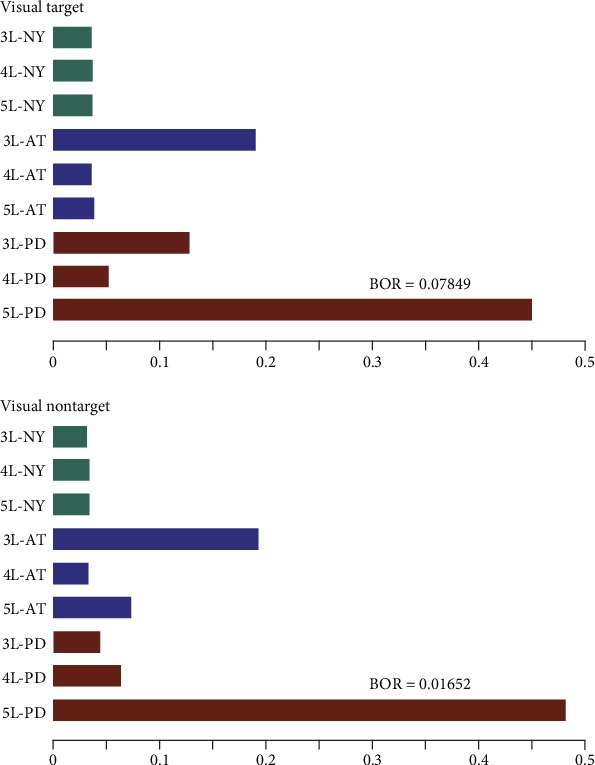
Results of group study shown as the expected posterior probability and Bayesian omnibus risk assessed by each testing scenario and MSP that is employed as ESI solution.

Figure 7 shows that the best brain structure is 5L-PD that certainly achieves the highest expected posterior probability (the top bar highlighted with red) with high confidence since the BOR value is much lower than 0.25. The second-highest performance is achieved by the 3L-AT testing scenario, outperforming even the 3L and 4L layer arrangements of the PD models. Further, there is no apparent benefit in selecting one of the remaining scenarios of PD or AT models. In fact, except in the case of the 5L configuration, either model behaves similarly. Nonetheless, all scenarios that have been tested for the NY-based brain tissue models reach the lowest probability of a model generating the observed data, confirming that this is the worst strategy of incorporating anatomical priors.

Regarding the used tissue model complexity, the performed Bayesian model selection only infers that the five-layer arrangement provides the most substantial source reconstruction performance. Other patterns of complexity tissues are comparable regardless of the used stimuli and combined head model to integrate the brain structure priors.

**Figure 8:**
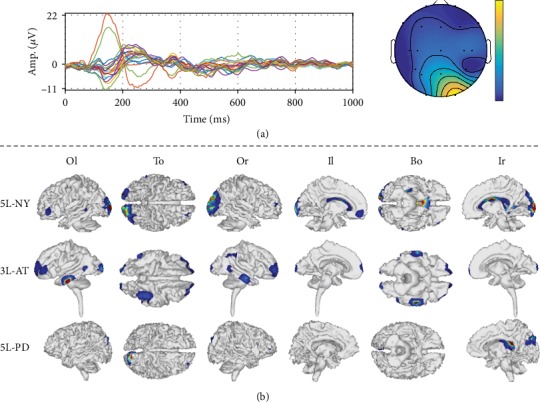
ESI solution for a representative subject using the best-achieved head models for each structural prior information, namely, 5L-PD, 3L-AT, and 5L-NY. (a) Sensor space: ERP and topographic map. (b) Reconstructed activity. Views: Or: outside right; Ol: outside left; To: top; Bo: bottom; Ir: inside right; Il: inside left.

To check the quality achieved by each tested ESI solution, we provide a visual inspection of the obtained source reconstruction for a representative subject, using the best-achieved head model for each tested scenario, i.e., 5L-PD, 3L-AT, and 5L-NY. Thus, Figure 8 shows the sensor space data, namely, an example of ERP and scalp topography, as well as the results of the source reconstruction (dipole-wise power). Both scalp topography and source activity are averaged in the time range from 100 to 200 ms (typically termed the N200 component), which contains brain-specific responses related to the processing of visual stimuli [41]. The ERP waveform shows a prominent temporooccipital negative peak close to 180 ms, which is mostly related to visual processing.

Moreover, the reconstructed activity localizes some components in the vicinity of the temporal lobe, covering the visual cortices for all the tested models. However, the 5L-NY model spreads the brain activity, which makes some activations appear in the nonvisual-related areas. 3L-AT activates the middle temporal gyrus that has some visual-related tasks. Additionally, in 5L-PD, an activity patch appears over the posterior cingulate gyrus, which occurs when a high demand for visual processing/discrimination is required, confirming that this kind of prior enhances the reconstruction of neural activity.

**Figure 9:**
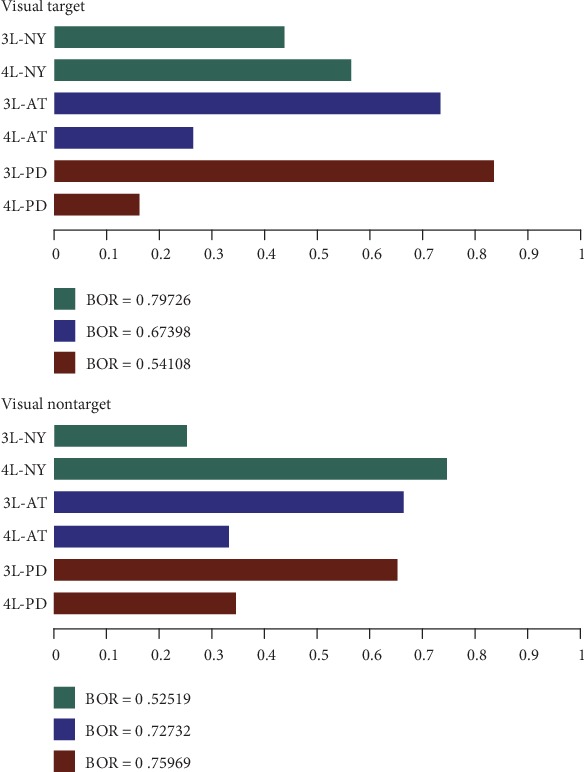
Expected posterior probability and Bayesian omnibus risk for the group study of 3L and 4L scenarios for each case of image data.

Another aspect to consider is the accuracy of brain activity source localization achieved by the volumetric brain segmentation that we model through the number of tissues incorporated into the head model. From the results performed by MSP, Figure 9 shows that including CSF segmentation (i.e., 4L**)** outperforms the conventionally used three-layer head model just in the case of NY tissue morphology. Otherwise, the discrepancy in performance between both configurations may vary from case to case.

Lastly, we investigate the distinction between the white matter and the gray matter in the volumetric brain segmentation, proving that the highest model complexity (5L) performs almost the best for each method which includes the anatomical priors regardless the elicited stimulus (see Figure 7).

## 5. Discussion

To enhance the EEG source localization, we develop an individually defined forward modeling that includes a subject-specific cortical mesh and also enhances the model of the brain structure, including prior knowledge of the tissue morphology and head segmentation complexity. Through the above-validated results on real EEG/ERP data, the following findings are worth mentioning.

### 5.1. Contribution of Anatomical Structure Priors

For incorporating anatomical priors of the brain tissues in the EEG forward problem, the radar charts show that the patient-dependent head model is the best-considered head model that is followed by the AT models, with the NY model being the worst representation. This result agrees with the commonly reported criterion of modeling structure priors, stating that the more the individual anatomical information, the smaller the source localization error. However, modeling of the brain tissues is very sensitive to wrong shape approximations of the target population (form, size, and demographics), which lead to significant errors of source localization. Thus, we are not aware of any demographic details on the widely used NY head model, and consequently, it is very likely to be far more representative of any particular target population. For reducing the shape differences between modeled and actual head tissues, the AT model builds an averaged atlas extracted from a population as similar as possible to the target. Another aspect to consider is the variability observed between the examined patient groups, as shown by the radar charts in Figure 4. Hence, some incongruent approximations may appear, making the brain structure deteriorate due to the head segmentation complexity (see Figure 5). As a result, the patient-specific head model that provides the inclusion of more realistic knowledge of the brain tissue morphology allows the most increase of the performance of EEG source localization.

### 5.2. Impact of Volumetric Brain Segmentation

In particular, we investigate the inclusion of the CSF compartment that has been reported as having an impact on the source reconstruction. However, we obtain that the discrepancy in performance between neglecting CSF and adding this tissue to the conventionally used configuration may vary from case to case. The drop in performance may be explained because some small changes in CSF layer thickness can provide a significant effect on EEG signal magnitudes in several standard visual paradigms [42]. Instead, the distinction between the white matter and the gray matter makes the volumetric segmentation perform almost the best for each method including the anatomical priors, demonstrating that neglecting this tissue division diminishes the ESI solution results remarkably [43]. So the volumetric brain segmentation influences strongly on the forward and inverse problems, but it must be carried out as accurately as possible.

Due to accurate volumetric tissue segmentation rules on the construction of a realistic finite element head conductivity for EEG source localization, the intended forward methodology must be adaptable to the voxelized structural information provided by the volumetric segmentation, including more complex tissues. In particular, we use the FDRM method that fulfills the needed conditions at a negligible computational effort.

### 5.3. The Benefit of Individually Defined Source Reconstruction

More realistic modeling of the sulci and gyrus of the brain enhances the individually defined forward model. In practice, canonical source reconstruction is employed that pools anatomical data from multiple subjects, being very susceptible to intersubject variability in cortical anatomy that poses a factor of an inaccurate tissue modeling. To cope with this issue, we propose a cortical mesh that is transformed anatomically to produce the subject-specific mesh. For this, we confine all sources of brain electrical activity within the gray matter by applying a dilation-based morphological operator over the volumetric segmentation of white matter, resulting in a segmented middle area between the cortex and WM boundary. As an additional advantage, our source space generation proposal is easily adaptable to a wide class of prior structural information as it does not require any manual intervention for ensuring that sources are enclosed within the gray matter. As a concrete example, Figure 8 shows some focalized sources in the posterior cingulate gyrus, which in fact must be related to the tested, visual stimulus [44]. Therefore, even if the patient-dependent approaches demand the cortical surface extraction from an individual's MRI, an efficient method for computing the EEG forward problem, along with the head model, is essential to provide more accurate localization of very focal sources.

As regards the influence of the used dipole source localization, we also compare two well-established approaches: LORETA and MSP. The former ESI approach reconstructs mostly superficial sources, favoring the simplest head model (i.e., the NY template) that produces the poorest detailed description of the tissue structures. Instead, the MSP method enables the reconstruction of superficial as well as deep sources, embracing the entire cortical surface. As a result, a more elaborate ESI solution also enhances the individually defined source reconstruction.

## 6. Conclusion

We discuss the improvement of EEG source localization performance by combining the head geometry complexity of the modeled tissue compartments and the prior knowledge of the brain tissue morphology, attempting to enhance the brain structure model in the individually defined forward problem formulation. Several testing scenarios for EEG source imaging are performed on 25 children, from which a set of MRI scans is acquired for extracting the anatomical priors and an EEG/ERP set for validating the EEG source imaging scenarios. For model comparison, the more probable model to generate the available data is determined by Bayesian log evidence.

As a result, only enough accurate volumetric brain segmentation can efficiently influence the forward problem, at least, for the examined cases of CSF, WM, and GM tissue segmentation, allowing identifying neural disorders and abnormalities. In turn, the complementary use of a patient-specific head model, as a strategy to personalize the head models in the forward problem, allows steadily increasing the source reconstruction performance in comparison to the contrasted atlas and template-based head models. Therefore, even if the patient-dependent approaches demand the cortical surface extraction from an individual MRI, an efficient computing of the individually defined forward problem is essential to provide more accurate localization of quite focal sources.

We develop an approach that automatically defines the source space in the gray matter volume, without any manual intervention, and regardless of the provided anatomical priors. Furthermore, the incorporation of patient-specific head models places all defined sources inside the patient-specific brain cavity, improving the localization of focal and deep sources.

Another aspect to consider is the influence of the used source localization, for which we compare two well-established approaches (LORETA and MSP), obtaining that a more elaborate ESI solution also enhances the individually defined source reconstruction. However, the tissue combination must be defined according to the used source localization method. On the one hand, the simplest tissue model encourages recovering superficial sources, benefiting the LORETA performance. On the other hand, a more complex tissue combination enhances the localization of focal and deep sources, improving the MSP performance.

Since the discussed methodology uses a set of fixed dipole orientations perpendicular to the brain surface and isotropic tissue conductivities, as future work, we plan to generate a forward model with anisotropic tissue conductivities and to develop a methodology that allows estimating the dipole amplitude and orientation. Additionally, we aim to test the ESI performance when more tissues such as eyes or fatty tissue are included. Also, even if previous investigations show that CSF is crucial to enhance the ESI accuracy, the finding that adding CSF does not help with accuracy in PD models prompts us to analyze more profoundly the influence of the particular database used in our study.

## Data Availability

The brain image data acquired from children and used to support the findings of this study are available from the corresponding author upon request.
